# The Endometriosis Impact Questionnaire (EIQ): a tool to measure the long-term impact of endometriosis on different aspects of women’s lives

**DOI:** 10.1186/s12905-019-0762-x

**Published:** 2019-05-14

**Authors:** Maryam Moradi, Melissa Parker, Anne Sneddon, Violeta Lopez, David Ellwood

**Affiliations:** 1 Nursing and Midwifery Care Research Center, Mashhad University of Medical Sciences, Mashhad, Iran; 2 Endometriosis Clinic, Canberra Hospital, Canberra, Australia; 3 Griffith University, School of Medicine, Queensland, Australia; 4 Yong Loo Lin School of Medicine, National University of Singapore, Singapore, Singapore

**Keywords:** Endometriosis, questionnaire, psychometrics, reliability, validation study, quality of life

## Abstract

**Background:**

Endometriosis is a chronic disease impacting on many aspects of a woman’s life. Because of the chronic and recurring nature, many of the impacts of endometriosis could be missed using existing questionnaires which focus on recent events. Therefore, a questionnaire with a long-term perspective is necessary. This study aimed to develop and evaluate a questionnaire to measure the long-term impact of endometriosis on different aspects of women’s lives.

**Methods:**

Through a methodological design, phase 1 was qualitative and phase 2 was a cross-sectional study. The original 100 EIQ items were developed based on results from an earlier qualitative study and literature review. Through a process of assessing face and content validity this was reduced to 66 items. The psychometric properties of the final 63 item EIQ were evaluated through a web-based survey with data from 423 responders with a self-reported surgically-diagnosed endometriosis.

**Results:**

Participants were aged 16-58 years. Exploratory factor analysis of a 66-item EIQ was established with 423 responders. The final 63-item EIQ contained six dimensions including: 33-item physical-psychosocial; 3-item fertility; 7-item sexual; 11-item employment; 6-item educational; and 3-item lifestyle. Cronbach’s alpha of 0.99 for the whole 63-item EIQ, and 0.84 to 0.98 for the dimensions suggests a very good reliability. High positive correlations between the EIQ and the EHP-5 (altered recall period) indicated good evidence of concurrent validity. High intra-class correlations indicated very good test-retest reliability.

**Conclusions:**

The EIQ, as a disease-specific questionnaire, could be used to provide a better understanding of the impact of endometriosis on different aspects of life, to better meet the needs of women. We recommend additional studies to establish validity evidence for the EIQ, including studies in other countries and languages.

**Electronic supplementary material:**

The online version of this article (10.1186/s12905-019-0762-x) contains supplementary material, which is available to authorized users.

## Background

Endometriosis is a high prevalence condition causing chronic pelvic pain, and the third leading cause of gynaecological hospitalization in the United States with high rates of hysterectomy [1]. Endometriosis requires lifelong management [2] and treatment must be individualized, taking into account the entire clinical problem, including disease impact and effects of treatment on quality of life (QoL) [3]. The most widely used instruments are the generic Health-Related Quality of Life (HRQoL) questionnaire including the Short Form–36 version (SF-36) [4, 5], the short version of the World Health Organization QoL (WHOQOL-BREF) [6, 7], and the EuroQol-5D [8]. However, the general HRQoL questionnaires do not consider unique variables related to endometriosis such as infertility [9] and the impact of symptoms other than pain. There are some specific questionnaires including Endometriosis Health Profile-30 (EHP-30) [10] and its subset, the EHP-5 [11], Endometriosis Treatment Satisfaction Questionnaire (ETSQ) [12], daily electronic Endometriosis Pain and Bleeding Diary (EPBD) [13], and ‘Patient-Centred Endometriosis’ questionnaire of Endo Care Questionnaire (ECQ) [14] but the EHP-30 is currently the disease-specific questionnaire to measure the HRQoL with the strongest validity evidence [9].

Published studies assessed the impact of endometriosis on pain using the VAS, depression using the BDI or HAM-D, anxiety using STAI or HAM-A [7], work impairment using WPAI [5, 8], sexual satisfaction using GRISS [6] and the GSSI [15], and sexual function using the FSDS or FSFI [16], and DSFI [15]. There is limited research on the psychological impacts of endometriosis except for depression/anxiety, work, education, social life, and lifestyle.

Existing tools only asses a subset of QOL indicators, and measure disease impact with a perspective of four weeks or less. Due to the chronic, recurring nature of endometriosis, many disease impacts could be missed, so a multi-dimensional questionnaire with a longer-term perspective is necessary to provide a better understanding. Women’s perceptions of the impact of endometriosis have been explored through our earlier qualitative study [17]. We have now developed and evaluated the psychometric properties of a questionnaire to measure the longer-term impact of endometriosis on different aspects of women’s lives.

## Methods

**Fig. 1:**
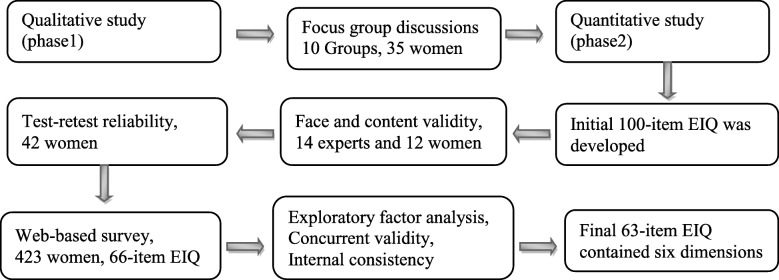
Development process of the EIQ

The Endometriosis Impact Questionnaire (EIQ) is a self-report questionnaire which asks women how endometriosis has affected their lives over the three recall periods including ‘last 12 months’, ‘1 to 5 years ago’ and ‘more than 5 years ago’. Categorical responses for all EIQ items are ranked using a 5 point Likert scale including: 0 = Not at all, 1 = A little, 2 = Somewhat, 3 = Quite a lot, 4 = Very much and 9 = Not applicable. Each item contributes equally and higher scores indicate a greater impact. The EIQ was developed and a psychometric evaluation conducted, using face, content, construct (factor analysis), concurrent validity, and reliability (internal consistency and test-retest reliability). The study used a methodological design which involved the development and evaluation of data collection instruments, scales or techniques [18]. To evaluate construct and concurrent validity and reliability, a cross-sectional study was conducted via a web-based survey. The development process is illustrated in Figure 1. All data were analyzed using SPSS version 20, and a probability values of p< 0.05 were considered to be statistically significant.

### Developing the items of the EIQ

The original 100 EIQ items were developed from a qualitative study [17] and from a systematic literature review. Eight steps on scale development [19], and the criteria for selecting items [20], were followed. The readability of the EIQ based on the Fog index [21] and ‘Readability Formulas’ [22] were used. In addition, endometriosis patients and experts were asked to evaluate readability of items [21].

### Evaluating the psychometric properties

#### Validity

The adequacy of a scale as a measure of a specific variable is an issue of validity [19]. The initial 100-item EIQ was evaluated to delete repeated items or those which related to only a few women from the focus groups discussions [17], so it was reduced to 89 items.

#### Face validity

Feedback was sought from 12 patients and 14 health professionals with expertise in endometriosis, questionnaires development, chronic disease, and psychology. They reviewed the 89-item EIQ and answered three open questions; *1. Does this appear to be a good measure of the impact of endometriosis? 2. List any areas pertinent to the impact of that are not covered 3. Share with us your feedback or any other comments to improve the EIQ.*

#### Content validity

For the content validity index (CVI) [21], we asked the same 12 patients and 14 health professionals to review the 89-item EIQ and rate the items based on ‘Relevance’, ‘Clarity’ and ‘Simplicity’ on a four-point scale. The CVI was measured as a percentage of the items rated as 3 or 4 [23, 24]. Item-level content validity index (I-CVI) was computed as the number of experts giving a rating of either 3 or 4, divided by the total number of experts [25]. A CVI or S-CVI of 0.80 was considered as indicating acceptability [23]. Following these steps the EIQ was reduced to 66 items.

#### Construct validity

The initial EIQ had randomly-ordered items to avoid hypothesised factor structure. But considering the number of items and the three recall periods, random-ordering made the EIQ difficult and time consuming to complete. Some experts suggested arranging the items in logical groups rather than having the items all mixed up, to make it easier to complete by responders. Therefore, the initial EIQ with randomly-ordered items was divided into logical groups based on categories which emerged from results of an earlier qualitative study [17], but the main categorizing of the EIQ was made based on exploratory factor analysis. The 66-item EIQ was then subjected to exploratory factor analysis to assess construct validity [26]. The commonly used method of principal component analysis and varimax rotation was used [27]. For this study, the acceptable level for factor loading was equal to or greater than 0.40 [28], and eigenvalues greater than one [27] were considered. The required sample size was estimated at 350 to 400 to have subject-to-variable ratio of 5:1, which identified as adequate in most cases [26].

#### Concurrent validity

There is no comparable questionnaire with a long-term perspective so to assess concurrent validity the EHP-5 [11] was chosen and the recall period changed from the ‘last 4 weeks’ to ‘last 12 months’.

#### Reliability

Two frequently used indicators of a scale’s reliability, including internal consistency (Cronbach’s alpha) and stability (Pearson correlation coefficient (r), test-retest reliability) were evaluated [26].

#### Internal consisten3cy reliability

Strong item-total correlations were identified as above 0.30 and inter-item correlations as ranging 0.30-0.70 [29]. In this study, a Cronbach’s alpha ≥ 0.7 was considered satisfactory and a corrected item-to-total correlation of at least 0.3 was considered as acceptable.

#### Test-retest reliability

To determine test-retest reliability, two copies of the final version were posted to 70 patients, who completed a first copy on the day that they received it, and a second copy after two weeks. Statistical differences between the two scores were assessed.

## Sample and setting

Convenience and snowball sampling were used to recruit a sufficiently large number of accessible participants, with an emphasis on those residing in Australia. Inclusion criteria were self-reported surgically-diagnosed endometriosis and ability to understand English. A web-based survey was conducted and recruitment was through different strategies from secondary or tertiary care levels, as well as from the general community. The EIQ-link was widely advertised via flyers disseminated or posted. Many groups and people (like endometriosis centres and endometriosis support groups) were asked to assist by forwarding an invitation email to their databases and/or by putting flyers in their centres or on their website/Facebook pages.

### Pilot study

A pilot study was conducted for the EIQ paper version and then for the EIQ online version. The EIQ paper version was finalized and pilot tested with eight patients in July 2013 and all responders provided positive feedback, stating that “It is comprehensive” and “It is clear”. The online EIQ was developed using ANU Polling Online (APOLLO) in August 2013 (Link: https://apollo.anu.edu.au/default.asp?pid=7700) and was pilot tested with four patients in September 2013. Feedback was generally positive stating that “It worked fine” and “The instructions are very clear”.

## Results

### EIQ item generation

The first draft of the EIQ included 100 items which reduced to 89. Face validity, content validity and further revisions resulted in a decrease to 66 items, and this version was used to assess factor analysis, concurrent validity, and test-retest reliability. Through factor analysis, three items were deleted and 63 items formed the final EIQ (Additional file 1).

### Demographic and clinical characteristics of responders

A total of 484 responders completed the final 63-item version between September 2013 and February 2014. One was excluded for providing incorrect completion; all 483 remaining questionnaires were fully completed. A further 60 responders were excluded as they did not have surgically-diagnosed endometriosis. Data from the remaining 423 responders were used to assess the psychometric properties of the EIQ. Demographic and clinical characteristics are provided in Table 1.

**Table 1:** Demographic and clinical characteristics of women with endometriosis (*n*=423)

Characteristics		*n* (%)
Age^a^ (years)		32.64± 8.38, (Range: 16-58)
Age groups	Aged 16-24	75 (17.73)
	Aged 25-34	186 (43.97)
	Aged 35 and above	162 (38.30)
Country of birth	Australia	313 (74.00)
	Other	110 (26.00)
Country at time of doing the EIQ	Australia	351 (82.98)
	Other	72 (17.02)
Language is spoken at home	English	418 (98.82)
	Other	5 (1.18)
Marital status	Married	187 (44.21)
	In a relationship	129 (30.50)
	Never married	48 (11.35)
	Single	45 (10.64)
	Separated/Divorced	13 (3.07)
	Widowed	1 (0.24)
Educational level	Primary or high school	14 (3.31)
	Lower secondary school	22 (5.20)
	Upper secondary school	47 (11.11)
	Vocational (e.g. TAFE)	79 (18.68)
	Some college/university	42 (9.93)
	Tertiary, undergraduate	131 (30.97)
	Tertiary, postgraduate	76 (17.97)
	Other	12 (2.84)
Employment^b^	Not employed	43 (10.17)
	Paid work, full time	200 (47.28)
	Paid work, part time	119 (28.13)
	Home duties	47 (11.11)
	Student, full time	30 (7.09)
	Student, part time	22 (5.20)
	Retired	3 (0.71)
	Other	38 (8.98)
Pregnancy history^b^	Never pregnant	237 (56.03)
	One or more children	126 (29.79)
	Miscarriage or stillbirth	64 (15.13)
	Currently pregnant	10 (2.36)
Delayed fertility	Not applicable (never tried)	194 (45.86)
	No	69 (16.31)
	Yes	160 (37.83)
Age at onset of symptoms^a^ (years)		16.35± 5.21, (Range: 8-42)
Age at first visit to doctor^a^ (years)		19.70±6.49, (Range: 9-43)
Age at diagnosis^a^ (years)		24.65± 6.58, (Range: 12-43)
Delay in diagnosis^a^ (years)		8.31±6.58, (Range: 0-28)
Endometriosis-related symptoms^b^ (life time).	Period pain	420 (99.29)
	Fatigue	397 (93.85)
	Bloating	386 (91.25)
	Pelvic pain not related to periods	382 (90.31)
	Ovulation/mid cycle pain	377 (89.13)
	Pain during/after sex	354 (83.69)
	Heavy bleeding	345 (81.56)
	Irregular bleeding	271 (64.07)
	Delayed fertility	158 (37.35)
	Other	91 (21.51)
Endometriosis treatments^b^ (life time)	Pain killers	410 (96.93)
	Surgical treatments	394 (93.14)
	Hormonal medications	368 (87.00)
	Complementary treatments	208 (49.17)
	Hormonal IUD	175 (41.37)
	Psychologist	120 (28.37)
	Nutritionist	95 (22.46)
	Physiotherapist	72 (17.02)
	Sexual therapist	15 (3.55)
	Other	48 (11.35)
Times presented to emergency department due to endometriosis	Never	165 (39.01)
	1-2	118 (27.90)
	3-4	55 (13.00)
	5-10	44 (10.40)
	More than 10	41 (9.69)
Hysterectomy due to endometriosis	No	376 (88.89)
	Yes	47 (11.11)

^a^Mean±SD

^b^Participants were asked to tick all that apply

### Readability of the EIQ

Gunning Fog Index was 8.7 for the 66-item EIQ and 8.6 for the 63-item EIQ, both of which are considered as ‘fairly easy to read’. Based on seven readability formulae the EIQ was scored as grade level 6, which means a standard/average reading level suitable for readers aged 10-11 years (fifth and sixth graders).

#### Face validity

Most believed that the EIQ was ‘comprehensive’ and a ‘good’ measure of the impact of endometriosis. Only one patient and four of the experts reported that the 89-item EIQ was too long and suggested reviewing it for redundant items.

#### Content validity

Content validity of the 89-item EIQ was based on seven out of 14 experts and six out of 12 patient’s ratings. The inter-rater agreements were 0.84 (experts) and 0.93 (patients). S-CVI and I-CVIs indicated acceptable content validity for both the EIQ and its items. Although, based on I-CVIs only two out of 89 items required deletion or change, a further 21 items were deleted to shorten to 66 items; these were items that overlapped, or had a low score on clarity or simplicity.

#### Construct validity

A sample size of 423 was adequate for factor analysis based on a Kaiser-Meyer-Olkin (KMO) test result of more than 0.9 and a Bartlett’s test of sphericity that was significant (p < 0.01). An inspection of the scree plot revealed a break after six or seven factors. All items with a factor loading of 0.4 and above were retained. The results from the factor analysis of the EIQ and each of three recall periods including ‘last 12 months’, ‘1 to 5 years ago’ and ‘more than 5 years ago’ were consistent to support having six separate factors for physical-psychosocial, fertility, sexual, employment, educational and lifestyle. The exception was that in the ‘more than 5 years ago’ period some of the physical items were loaded into a separate factor. However, to have consistent factor dimensions, it was decided to include the physical items into the physical-psychosocial dimension. Factor analysis with six factors explained 58.14, 53.84, 52.86, and 46.09 of the total variance in recall period of ‘last 12 months’, ‘1 to 5 years ago’, ‘more than 5 years ago’, and the total EIQ respectively. Three items were removed from the 66-item EIQ because they did not reach a 0.4 factor loading. In comparison with the primary EIQ categorizing, factor analysis identified a new dimension which was called fertility, combined the physical, psychological and social items into one factor called physical-psychosocial, and transferred some items between dimensions.

#### Final EIQ

The final EIQ with 63 items had six dimensions: (1) physical-psychosocial - 33 items (consisting of physical – 13 items, psychological- 16 items and social impact – 4 items); (2) fertility - 3 items; (3) sexual - 7 items; (4) employment - 11 items; (5) educational - 6 items; and (6) lifestyle - 3 items (Additional file 1).

#### Concurrent validity

There were statistically significant high positive correlations (*r=0*.66-0.80, (n=423), p < 0.01) between the ‘last 12 months’ of the EIQ and the EHP-5 (Table 2), indicating that patients who experienced a greater impact (higher EIQ score) had worse health-related quality of life (higher score from the EHP-5). Therefore, the recall period of ‘last 12 months’ for the EIQ, had high concurrent validity or very good correlation with the EHP-5. Correlations were good (*r* = 0.40-0.58) when the EHP-5 was compared with the ‘1 to 5 years ago’ period of the EIQ, and medium or low (*r =* 0.17-0.45) when compared with the ‘more than 5 years ago’ period of the EIQ, except for sexual dimension and intercourse module, which was good at a level of p= 0.45.

**Table 2:** Concurrent validity correlations of the ‘last 12 months’ EIQ dimensions with the EHP-5 scales (*n* = 423)

EIQ (last 12 months)	EHP-5	Number	Pearson Correlation^a^
Physical-psychosocial	EHP-5 (score from 5 core items)	412	.80
Physical	Pain	407	.66
Psychological	Q2,3, & 5 of the core questionnaire^b^	409	.75
Social	Social support	402	.64
Sexual	Intercourse	363	.71
Fertility	Infertility	348	.66
Employment	Work	360	.66

^a^All Correlations were significant at the 0.01level (2-tailed) using Pearson Correlation

^b^Control & powerlessness, emotional wellbeing and self-image

#### EIQ Scoring

The score for each dimension at each recall period was the sum of all applicable items divided by the maximum score, rescaled to 0-100. The total score for each dimension was calculated as a mean of the three recall periods. If responders skipped a dimension or recall period, because they were not applicable to them, a score for that dimension or recall period could not be calculated.

An SPSS code was provided to score the EIQ. The formula for scoring the EIQ and its dimensions on a scale from 0-100 was the sum of the scores of the applicable items multiplied 100/the maximum score of the applicable items, which was the number of applicable items x 4. The dimensions were scored on a scale from 0 (minimum possible impact), to 100 (maximum possible impact) as measured by the EIQ.

The EIQ scores for the sample as a whole, and the distribution of the scores are reported in Table 3. At each recall period, and also over a total of three recall periods, the highest impact of endometriosis was on fertility followed by the physical-psychosocial dimension, and the lowest impact was on the lifestyle dimension.

**Table 3:** Descriptive statistics of the EIQ dimension scoring

EIQ dimensions	Last 12 months	1 to 5 years ago	More than 5 years ago	Total three recall periods
Physical-Psychosocial				
Mean ± SD	62.69 ± 27.25	64.27 ± 23.90	56.12 ± 24.90	61.10 ± 21.28
Number ^a^	412	418	401	390
Minimum (%)	.00 (1.9)	.00 (1.4)	.00 (.9)	6.31 (.9)
Maximum (%)	100.00 (1.9)	100.00 (1.9)	100.00 (1.4)	100.00 (1.4)
25	44.70	50.00	38.11	46.81
Percentile 50	68.56	68.94	58.00	63.52
75	85.61	82.58	75.38	77.28
Physical				
Mean ± SD	62.46 ± 27.38	64.46 ± 23.61	58.93 ± 24.50	61.88±20.51
Number	407	416	400	384
Minimum (%)	.00 (2.6)	.00 (2.1)	.00 (.9)	8.97 (.2)
Maximum (%)	100.00 (4.3)	100.00 (4.0)	100.00 (3.5)	100.00 (1.4)
25	46.15	51.92	40.91	47.09
Percentile 50	68.75	67.31	61.54	63.46
75	84.62	81.25	76.92	76.28
Psychological				
Mean ± SD	65.00 ± 27.89	66.21 ± 25.07	57.26 ± 27.31	62.76 ± 22.89
Number	409	416	389	375
Minimum (%)	.00 (1.9)	.00 (2.4)	.00 (3.5)	1.56 (.2)
Maximum (%)	100.00 (4.0)	100.00 (3.8)	100.00 (3.3)	100.00 (1.7)
25	48.44	50.00	39.06	46.15
Percentile 50	71.88	72.60	59.38	67.19
75	87.50	84.38	78.59	79.69
Social				
Mean ± SD	54.81 ± 34.89	56.01 ± 32.20	47.23 ± 32.67	52.44 ± 29.09
Number	402	407	373	352
Minimum (%)	.00 (11.3)	.00 (8.5)	.00 (11.6)	.00 (4.3)
Maximum (%)	100.00 (16.8)	100.00 (13.9)	100.00 (10.6)	100.00 (5.9)
25	25.00	31.25	18.75	29.17
Percentile 50	62.50	56.25	43.75	54.17
75	87.50	81.25	75.00	76.56
Sexual				
Mean ± SD	47.24 ± 28.65	47.87 ± 27.93	40.88 ± 29.89	45.53 ± 26.77
Number	375	381	343	320
Minimum (%)	.00 (3.5)	.00 (3.8)	.00 (1.9)	.00 (.2)
Maximum (%)	100.00 (5.4)	100.00 (3.8)	100.00 (1.9)	100.00 (2.4)
25	25.00	25.00	44.70	22.92
Percentile 50	50.00	46.43	68.56	44.05
75	67.86	71.43	85.61	67.86
Fertility				
Mean ± SD	70.23 ± 37.59	67.78 ± 36.86	58.24 ± 38.90	65.28 ± 32.70
Number	391	390	347	323
Minimum (%)	.00 (13.2)	.00 (11.3)	.00 (14.7)	.00 (4.0)
Maximum (%)	100.00 (46.1)	100.00 (41.1)	100.00 (27.0)	100.00 (18.9)
25	37.50	37.50	25.00	33.33
Percentile 50	91.67	83.33	66.67	75.00
75	100.00	100.00	100.00	97.22
Employment				
Mean ± SD	43.89 ± 34.77	43.66 ± 32.78	35.24 ± 32.40	41.04 ± 30.42
Number	385	392	357	334
Minimum (%)	.00 (10.2)	.00 (6.4)	.00 (11.3)	.00 (2.4)
Maximum (%)	100.00 (9.0)	100.00 (6.9)	100.00 (5.2)	100.00 (3.5)
25	11.36	15.91	8.71	14.20
Percentile 50	36.36	34.69	25.00	32.95
75	75.00	72.67	60.56	64.58
Educational				
Mean ± SD	46.61 ± 39.32	45.48 ± 35.76	43.99 ± 33.71	43.78 ± 31.28
Number	173	206	241	149
Minimum (%)	.00 (10.2)	.00 (8.5)	.00 (6.6)	.00 (2.4)
Maximum (%)	100.00 (7.1)	100.00 (7.1)	100.00 (6.1)	100.00 (2.4)
25	2.08	8.33	12.50	16.67
Percentile 50	45.83	43.75	37.50	38.89
75	83.33	79.17	75.00	70.83
Lifestyle				
Mean ± SD	16.54 ± 23.88	20.48 ± 26.78	20.75 ± 28.76	19.43 ± 24.44
Number	391	397	367	342
Minimum (%)	.00 (46.6)	.00 (43.0)	.00 (42.6)	.00 (29.8)
Maximum (%)	100.00 (2.4)	100.00 (2.8)	100.00 (3.5)	100.00 (1.7)
25	.00	.00	.00	.00
Percentile 50	.00	8.33	8.33	8.33
75	25.00	33.33	33.33	33.33

^a^Number of responders for whom that dimension at that recall period was applicable. Regarding the total scores, if responders skipped one of the recall periods, the total score for that dimension was not calculated

The scores were calculated on a scale from 0-100; a higher score means a greater impact of endometriosis on that dimension of life. 0=Minimum impact of endometriosis as measured by the EIQ; 100= maximum possible impact of endometriosis as measured by the EIQ

Out of 423 responders, 26 (6.1%) participants skipped items from the sexual dimension, 26 (6.1%) participants skipped items from the employment dimension, 152 (35.9%) participants skipped items from the education dimension, and 8 (1.9%) participants skipped items from the fertility and lifestyle dimensions

#### Completion time of the EIQ

The estimated completion time for the 66 item EIQ was 4.8-31.5 minutes with a mean ± SD of 15.2 ± 6.2 minutes.

#### Reliability

The internal consistency reliability of the 63-item EIQ and the six dimensions was Cronbach’s alpha 0.98 (Table 4). Results showed good reliability in all dimensions but the physical-psychosocial dimension had the highest and the lifestyle dimension had the lowest Cronbach’s alpha for all three recall periods.

**Table 4:** Internal consistency of the 63-item EIQ and its dimensions

	Number^a^	last 12 months	1 to 5 years ago	more than 5 years ago"	Total three recall periods
EIQ total	423	.97 (63 items)	.97	.97	.98(189 items)
Physical-psychosocial	423	.97 (33 items)	.96	.95	.98 (99 items)
Physical	423	.92 (13 items)	.90	.90	.94 (39 items)
psychological	423	.95 (16 items)	.93	.92	.96 (48 items)
Social	423	.91 (4 items)	.90	.88	.93 (12 items)
Sexual	397	.84 (7 items)	.85	.86	.93 (21 items)
Fertility	415	.73 (3 items)	.73	.66	.85 (9 items)
Employment	397	.94 (11 items)	.94	.94	.97 (33 items)
Educational	271	.93 (6 items)	.92	.91	.93 (18 items)
Lifestyle	415	.53 (3 items)	.57	.62	.84 (9items)

Cronbach’s alpha was evaluated

^a^Only responders who skipped all items of the dimension (all three recall periods) were omitted

In the 63-item EIQ, all items correlated to total items at a level above 0.3 except for item numbered Q41, which correlated to total items at a level of 0.2. Cronbach's alpha for each recall period of the EIQ was 0.97 across the 63 items, indicating very good reliability. In each recall period, all items correlated to total items at a level above 0.3 except for two items, which correlated to total items at a level of 0.2. Within each dimension in each recall period, all items correlated to total items at a level above 0.3.

Results of the inter-scale correlation matrix is shown in Table 5. Only responders who answered for both dimensions were included. All correlations were positive and statistically significant and there were high correlations between most dimensions at all recall periods.

**Table 5:** Inter-scale correlation matrix for the last 12 months, 1 to 5 years ago, More than 5 years ago

	Physical-psychosocial	Sexual	Fertility	Employment	Educational	Lifestyle
Physical-Psychosocial	1.00					
Sexual	.63^a^	1.00				
	.64^b^					
	.63^c^					
Fertility	.41^a^	.29^a^	1.00			
	.37^b^	.24^b^				
	.47^c^	.33^c^				
Employment	.74^a^	.53^a^	.31^a^	1.00		
	.72^b^	.54^b^	.24^b^			
	.70^c^	.55^c^	.35^c^			
Educational	.59^a^	.43^a^	.16^a^	.72^a^	1.00	
	.60^b^	.47^b^	.20^b^	.69^b^		
	.62^c^	.45^c^	.27^c^	.70^c^		
Lifestyle	.37^a^	.32^a^	.13^a^	.31^a^	.30^a^	1.00
	.38^b^	.28^b^	.13^b^	.31^b^	.33^b^	
	.48^c^	.44^c^	.17^c^	.43^c^	.32^c^	

Pearson correlation coefficient (r) was evaluated

^a^Last 12 months: All Correlations were significant at the 0.01level (2-tailed) except for fertility-education and fertility-lifestyle, which were significant at the 0.05 level (2-tailed)

^b^1 to 5 years ago: All correlations were significant at the 0.01level (2-tailed) except for fertility-lifestyle, which was significant at the 0.05 level (2-tailed)

^c^More than 5 years ago: All correlations were significant at the 0.01level (2-tailed)

#### Test-retest reliability (stability)

Test retest reliability using intra-class correlation (ICC) was conducted in 40 out of 70 (60%) respondents who completed the second test after a two-week interval using the EIQ paper version. The results showed a statistically significant ICC between all dimensions at times one and 2, ranging from 0.88-0.99. The results indicate that the EIQ has very good test-retest reliability.

## Discussion

This study shows that the EIQ is a valid, reliable tool to measure the impact of endometriosis on women’s lives with a long-term view. Endometriosis is a chronic disease as symptoms may continue despite seemingly adequate treatment [30]. Considering its recurring nature, there are some impacts that could be missed by only looking at the last four weeks. For example, current questionnaires are not able to measure the impacts on a woman who lost her sexual-intimate relationship or who was not able to complete studies and/or work goals, loss of job or promotion opportunities, or addressed her regrets from living with endometriosis. These impacts were revealed during our earlier qualitative study [17] and are supported by others [31, 32]. The EIQ is the first questionnaire to measure multi-dimensional impacts with a long-term view.

Validation of a scale involves the collection of empirical evidence concerning its use^26^. Similar to the EIQ, the ETSQ [12] and the EPBD [13] were developed from focus group discussions and interviews with patients. Items of the EHP-30 were generated based on open-ended exploratory interviews with 25 women with endometriosis [10]. The process used to validate the EIQ was to some extent similar to the one employed for the ECQ [14], however the development processes were different.

Compared with the EHP-30 [10], the EIQ has two new subscales of education and lifestyle, whilst the EHP-30 has subscales of relationship with children, medical professionals, and treatment. Little is known about the impacts of endometriosis on lifestyle but negative impacts were reported during our earlier study [17]. Future studies should be conducted to measure the impact of endometriosis on lifestyle, as well as education and work which extend beyond time off and lost productivity addressed in previous studies [5, 8].

The demographic and medical characteristics of the responders in this study are consistent with endometriosis patients in previous research, increasing the generalizability of results. In a multinational study [5], 745 participants had a mean age of 32.5 years and a mean delay in diagnosis of 6.7 years, similar to the mean age (32.6) and delay (8.3) in this study. In an online survey most of 8008 patient (from China, France and Russia) were diagnosed within five years [33]. Thirty-eight percent of EIQ responders had delayed fertility, consistent with the infertility rate of 37% among 6,146 European patients [34] and 42.5% among 7,020 US patients [35]. Eleven percent had a hysterectomy because of endometriosis, which is within the range of 8-29% reported by others [36].

The self-reporting characteristic of the EIQ has pros and cons. Sensitive information is more frequently and accurately reported in self-administered modes than when interviewers ask the questions [37]. However, limitations related to reporting and recall errors may apply because of the self-reported and retrospective nature of the EIQ. Questionnaire developers can overestimate people’s ability to recall past events [20]. However, life time recall period has been used previously with satisfactory psychometric characteristics [14]. The decision to have three recall periods was based on focus group discussions where women reported fluctuations in the impacts, variety of symptoms at different times, and differing perceptions of impacts based on their situation, desires, responsibilities and plans [17]. It is acknowledged that using multiple time frames might complicate the questionnaire and overburden respondents but the completion time of the EIQ was reasonable.

### Applications of the EIQ

Use of disease-specific instruments in endometriosis research has been highlighted and preferred in recent literature reviews [9, 38]. The EIQ, as a disease-specific questionnaire, could be used to provide detailed information on the multi-dimensional impacts of endometriosis in population health surveys, to compare different areas and stages of patients’ lives or different management options. It could be used with all three recall periods or each period independently, because each has satisfactory validity and reliability. The total score for each dimension at three recall periods, for all dimensions at each recall period and the total impact score could be calculated. Combining the scores will depend on the research objectives. Using the recall period of ‘last 12 months’, could be used in clinical trials and to investigate outcomes. It could be useful to guide development of an individualized disease management plan, and could help patients to communicate with health professionals and contribute to such a plan. It could also be used as a burden-estimation or needs assessment tool to provide information for making health policy decisions to improve services.

### Strengths of this study

The EIQ was developed from focus group discussions, along with an extensive literature review, which ensured that the items are relevant to patients from either tertiary care or the community. A sample of 423, reflected 6.41 responders per item, is close to being a ‘very good’ sample size [39]. There were no missing data as most items were compulsory in the online EIQ, but even those who answered the paper version for the test-retest did not miss any questions. The online questionnaire facilitated access to women within and beyond Australia, which was time and expense-saving.

### Limitations of this study

Non-probability sampling decreases the ability to generalize results. Dissemination of the study link was focused inside Australia and 74% of responders to the online EIQ were born in Australia. Therefore, the applicability to Australian women with different characteristics to the participants, and non-Australians might be limited. Limitations related to reporting and recall errors may apply. As a web-based survey was used, the generalizibility of the results is restricted to those who are keyboard and Internet literate [40]. In addition, the current study did not collect clinical information regarding the severity of endometriosis lesions and the existence of comorbidities, such as mood disorders, obesity, musculoskeletal and neuropathic sources of pain, and patients undergoing treatment with psychotropic drugs that could also contribute to symptoms, and it is acknowledged that not knowing these clinical characteristics of the sample could limit the generalizability of the findings.

## Conclusions

The EIQ has been developed and validated to measure the impacts of endometriosis on different aspects of women’s lives with a long-term view. It can be used by researchers and clinicians to provide a better understanding of the impact of endometriosis on different aspects of life over time, and to meet the needs of women living with this condition. We recommend additional studies to establish stronger psychometric properties including known-groups validity and sensitivity to change for the ‘last 12 months’ section. Further validity evidence is also recommended in clinically relevant subgroups of endometriosis patients, such as women presenting for fertility evaluations versus women presenting with chronic pelvic pain, as well as endometriosis patients with the comorbid conditions. Studies in other countries and languages are recommended to make multinational studies possible.

## Additional file

Additional file 1: 63-item EIQ. (PDF 428 kb)

## Abbreviations

EIQ: Endometriosis Impact Questionnaire
HRQoL: Health-Related Quality of Life
QoL: Quality of life
