# Supplementary material for: The Endometriosis Impact Questionnaire (EIQ): a tool to measure the long-term impact of endometriosis on different aspects of women’s lives
Source: BMC Womens Health. 2019 May 14;19:64. doi: 10.1186/s12905-019-0762-x (PMC6518659; doi:10.1186/s12905-019-0762-x)
Supplement: Supplementary file 1 — 63-item EIQ. (PDF 428 kb) [file 12905_2019_762_MOESM1_ESM.pdf]

Endometriosis Impact Questionnaire (63-item EIQ)

© Moradi M., et al.

Maryam Moradi, Melissa Parker, Anne Sneddon, Violeta Lopez, David Ellwood

---

- The questions in the EIQ have been developed based on 10 focus group discussions with 35 women with endometriosis. EIQ aims to measure the long term impact of endometriosis on different aspects of women's lives.
- Your valuable participation will contribute towards better understanding of the impact of endometriosis on women's lives and to better meet the needs of women with this condition.
- Please be assured that all responses are strictly confidential. Only combined data from all the questionnaires will be reported.
- Completing this questionnaire is voluntary. By completing the questionnaire you are indicating your consent to participate in the study.
- There are no right or wrong answers to any of the questions, so please respond according to your feelings and experiences about how endometriosis has affected your life.
- This questionnaire takes about 15(± 6) minutes to complete.
- If you have any questions about the questionnaire or the study please contact Maryam Moradi by email maryam.moradi.fu@gmail.com.
- Once you have completed the questionnaire please return it to us in the envelope provided.

We appreciate you filling out all dimensions to help us complete this important study.

© Moradi M., et al.

Instructions: We ask you to complete the EIQ questionnaire by placing in every box the number which best describes how much endometriosis has affected your life over three time periods (last 12 months, 1 to 5 years ago and more than 5 years ago).

0 = Not at all
1 = A little
2 = Somewhat
3 = Quite a lot
4 = Very much
9 = Not applicable, was not relevant to you during that time period (e.g. did not have endometriosis in that time period or question is about the effect of endometriosis on working and you did not work in that time period)

Example:

**Because of My Endometriosis:**	**Last 12 months**	**1 to 5 years ago**	**More than 5 years ago**
**Q1. I had severe period pain.**	**1**	**3**	**4**
**Q2. I found it difficult to care for my child.**	**0**	**4**	**9**

This woman's answer for question 2 shows she did not have any children more than 5 years ago (9), then she had a baby 3 years ago and "Because of her endometriosis" she found it difficult "Very much" (4) to care for her child, but during the last 12 months she has not had this problem at all (0). Some dimensions of the EIQ may not relevant to you, so you could skip to the next dimension.

I have read the instructions and I am ready to begin. ☐ Yes

© Moradi M., et al.

Dimension 1. Physical Impact of Endometriosis (Questions marked * are mandatory)

Please complete questions by placing in every box the number which best describes how much endometriosis has affected your life. Please remember to put a number in all three columns.

0 = Not at all
1 = A little
2 = Somewhat
3 = Quite a lot
4 = Very much
9 = Not applicable, was not relevant to you during that time period (e.g. did not have endometriosis in that time period or question is not relevant to you)

Because of My Endometriosis:

		Last 12 months	1 to 5 years ago	More than 5 years ago
**Q1.***	I had severe period pain.			
**Q2.***	I had pelvic pain between my periods.			
**Q3.***	I had heavy bleeding with periods.			
**Q4.***	I had irregular spotting or bleeding between my periods.			
**Q5.***	I felt tired more than usual.			
**Q6.***	I spent time in bed or lying down due to pain (e.g. period or pelvic pain).			
**Q7.***	I had trouble sleeping.			
**Q8.***	I felt that my energy levels have decreased.			
**Q9.***	I had difficulties carrying out normal daily activities (e.g. shopping, driving).			
**Q10.***	I had to decrease my involvement in exercise or sport.			
**Q11.***	I was bothered physically by the side effects of medical or surgical treatment/s.			
**Q12.***	I was concerned about weight gain (e.g. due to less activity caused by pain or as a side effect of treatment).			
**Q13.***	I thought about having a hysterectomy to treat my symptoms.			

© Moradi M., et al.

Dimension 1. Psychological Impact of Endometriosis (Questions marked * are mandatory)

Please complete questions by placing in every box the number which best describes how much endometriosis has affected your life. Please remember to put a number in all three columns.

0 = Not at all
1 = A little
2 = Somewhat
3 = Quite a lot
4 = Very much
9 = Not applicable, was not relevant to you during that time period (e.g. did not have endometriosis in that time period or question is not related to you)

**Because of My Endometriosis:**		**Last 12 months**	**1 to 5 years ago**	**More than 5 years ago**
**Q14.***	I felt depressed.			
**Q15.***	I felt uncertain because of the unpredictable nature of endometriosis and its symptoms.			
**Q16.***	I felt uncertain about the effectiveness of my treatment/s.			
**Q17.***	I experienced mood swings (due to my symptoms/pain or treatment side effect).			
**Q18.***	I felt nobody understands how I feel.			
**Q19.***	I felt less self-confident.			
**Q20.***	I was unhappy about my appearance (e.g. due to weight gain, surgery scar/s).			
**Q21.***	I felt my identity has been disrupted as a woman, partner, mother etc.			
**Q22.***	I felt embarrassed (e.g. symptoms at work place, school, explaining to employers, colleagues or teachers).			
**Q23.***	I felt jealous (e.g. of others who have no pain or have had children or pain free sex).			
**Q24.***	I felt worried that my symptoms will get worse.			
**Q25.***	I was worried about the effect of endometriosis on my future plans.			
**Q26.***	I felt annoyed about the amount of painkillers I have had to take.			
**Q27.***	I had feelings of defeat or hopelessness (e.g. not being able to deal with this disease anymore).			
**Q28.***	I was concerned about overuse or accidental of pain killers.			
**Q29.***	I was not able to control my life as I would like.			

© Moradi M., et al.

Dimension 1. Social Impact of Endometriosis (Questions marked * are mandatory)

Please complete questions by placing in every box the number which best describes how much endometriosis has affected your life. Please remember to put a number in all three columns.

0 = Not at all
1 = A little
2 = Somewhat
3 = Quite a lot
4 = Very much
9 = Not applicable, was not relevant to you during that time period (e.g. did not have endometriosis in that time period or question is not relevant to you)

Because of My Endometriosis:

	Last 12 months	1 to 5 years ago	More than 5 years ago
**Q30.*** I reduced participation in social events like attending parties or going out with my friends.	☐	☐	☐
**Q31.*** I decreased my leisure activities (like hobbies or going on holidays).	☐	☐	☐
**Q32.*** I had problems with my relationships with other people (e.g. because of my mood swings or pain).	☐	☐	☐
**Q33.*** I felt isolated.	☐	☐	☐

© Moradi M., et al.

Dimension 2. Sexual and Intimate Relationships Impact of Endometriosis

Please complete questions by placing in every box the number which best describes how much endometriosis has affected your life. Please remember to put a number in all three columns.

0 = Not at all
1 = A little
2 = Somewhat
3 = Quite a lot
4 = Very much
9 = Not applicable, was not relevant to you during that time period (e.g. did not have endometriosis in that time period or you were not sexually active or partnered in that time period)

If all the following questions about "Sexual and Intimate Relationships Impact" are not relevant to you (never been sexually active or did not have endometriosis when you had sexual relationships) please tick the box and go to the next dimension. ☐

Because of My Endometriosis:

	Last 12 months	1 to 5 years ago	More than 5 years ago
**Q34**. I had pain during or after sexual activity.	☐	☐	☐
**Q35**. I had bleeding or spotting during or after sexual activity.	☐	☐	☐
**Q36**. I avoided sexual activity.	☐	☐	☐
**Q37**. I experienced strain in my relationship with my partner/s.	☐	☐	☐
**Q38**. I was not able to maintain long term relationships with my partner/s.	☐	☐	☐
**Q39**. I was not satisfied with my sex life.	☐	☐	☐
**Q40**. I had thoughts about being single due to sexual difficulties (e.g. pain or bleeding) or fertility issues.	☐	☐	☐

© Moradi M., et al.

Dimension 3. Fertility Impact of Endometriosis

Please complete questions by placing in every box the number which best describes how much endometriosis has affected your life. Please remember to put a number in all three columns.

0 = Not at all
1 = A little
2 = Somewhat
3 = Quite a lot
4 = Very much
9 = Not applicable, was not relevant to you during that time period (e.g. did not have endometriosis in that time period or question is not relevant to you)

If all the following questions about "Fertility" are not relevant to you please tick the box, and go to the next dimension. ☐

Because of My Endometriosis:

	**Last 12 months**	**1 to 5 years ago**	**More than 5 years ago**
**Q41**. I found it difficult to become pregnant.	☐	☐	☐
**Q42**. I was worried about my fertility.	☐	☐	☐
**Q43**. I had regrets about not being able to have a child/more children.	☐	☐	☐

© Moradi M., et al.

Dimension 4. Employment and Financial Impact of Endometriosis

Please complete questions by placing in every box the number which best describes how much endometriosis has affected your life. Please remember to put a number in all three columns.

0 = Not at all
1 = A little
2 = Somewhat
3 = Quite a lot
4 = Very much
9 = Not applicable, was not relevant to you during that time period (e.g. did not have endometriosis in that time period or did not study in that time period)

If all the following questions about "Employment" are not relevant to you (never worked or did not have endometriosis when you were working) please tick the box, **answer only Question 54** and go to the next dimension. ☐

Because of My Endometriosis:

	Last 12 months	1 to 5 years ago	More than 5 years ago
**Q44**. I had difficulty pursuing my preferred career.	☐	☐	☐
**Q45**. I experienced limitations in what I can do at work.	☐	☐	☐
**Q46**. I reduced my working hours.	☐	☐	☐
**Q47**. I took time off work.	☐	☐	☐
**Q48**. I experienced difficulty concentrating or focusing on my work.	☐	☐	☐
**Q49**. I think that I missed out on job promotions.	☐	☐	☐
**Q50**. I was afraid of losing my job.	☐	☐	☐
**Q51**. I had to change or give up my job.	☐	☐	☐
**Q52**. I had a reduction in my income.	☐	☐	☐
**Q53**. I felt that I was unable to reach my career goals.	☐	☐	☐
**Q54**. **I experienced financial hardship (due to the cost of diagnosis or treatment medications, surgery, infertility or lost job opportunities).**	☐	☐	☐

© Moradi M., et al.

Dimension 5. Educational Impact of Endometriosis

Please complete questions by placing in every box the number which best describes how much endometriosis has affected your life. Please remember to put a number in all three columns.

0 = Not at all
1 = A little
2 = Somewhat
3 = Quite a lot
4 = Very much
9 =Not applicable, was not relevant to you during that time period (e.g. did not have endometriosis in that time period or did not study in that time period)

If all the following questions about "Educational Impact" are not relevant to you (did not have endometriosis when you were at school/university) please tick the box and go to the next dimension. ☐

Because of My Endometriosis:

	Last 12 months	1 to 5 years ago	More than 5 years ago
**Q55**. I took time off school/studies.	☐	☐	☐
**Q56**. I experienced difficulty concentrating or focusing on my studies.	☐	☐	☐
**Q57**. I did not complete my study requirements on time.	☐	☐	☐
**Q58**. I missed school/university exams.	☐	☐	☐
**Q59**. I needed more time to complete schooling/studies (e.g. extensions, re-enrolment).	☐	☐	☐
**Q60**. I felt that I was unable to reach my education goals.	☐	☐	☐

© Moradi M., et al.

Dimension 6. Lifestyle Impact of Endometriosis (Questions marked * are mandatory)

Please complete questions by placing in every box the number which best describes how much endometriosis has affected your life. Please remember to put a number in all three columns.

0 = Not at all
1 = A little
2 = Somewhat
3 = Quite a lot
4 = Very much
9 = Not applicable, was not relevant to you during that time period (e.g. did not have endometriosis in that time period or question is not relevant to you)

**Because of My Endometriosis:**	**Last 12 months**	**1 to 5 years ago**	**More than 5 years ago**
**Q61.*** I consumed alcohol to help me cope (e.g. with my symptoms or feelings).	☐	☐	☐
**Q62.*** I smoked cigarettes (tobacco) to help me cope (e.g. with my symptoms or feelings).	☐	☐	☐
**Q63.*** I used other illicit substances or drugs to help me cope (e.g. with my symptoms or feelings).	☐	☐	☐

Comments (optional)

We welcome your comments about this questionnaire (EIQ), how to improve it and/or any further comments in relation to your experiences with endometriosis.

Thank you so much for your participation in this study.

You could contact Maryam Moradi, PhD candidate by email: maryam.moradi.fu@gmail.com for any concern regarding the questionnaire.

© Moradi M., et al.
